# Evaluation of Arm Swing Features and Asymmetry during Gait in Parkinson’s Disease Using the Azure Kinect Sensor

**DOI:** 10.3390/s22166282

**Published:** 2022-08-21

**Authors:** Claudia Ferraris, Gianluca Amprimo, Giulia Masi, Luca Vismara, Riccardo Cremascoli, Serena Sinagra, Giuseppe Pettiti, Alessandro Mauro, Lorenzo Priano

**Affiliations:** 1 Institute of Electronics, Computer and Telecommunication Engineering, National Research Council, Corso Duca degli Abruzzi 24, 10129 Torino, Italy; 2 Department of Control and Computer Engineering, Politecnico di Torino, Corso Duca degli Abruzzi 24, 10129 Torino, Italy; 3 Department of Neurosciences, University of Turin, Via Cherasco 15, 10100 Torino, Italy; 4 Istituto Auxologico Italiano, IRCCS, Department of Neurology and Neurorehabilitation, S. Giuseppe Hospital, Strada Luigi Cadorna 90, 28824 Piancavallo, Italy

**Keywords:** arm swing, gait analysis, Azure Kinect, Parkinson’s disease, spatiotemporal parameters, center of mass sway, asymmetry, movement analysis

## Abstract

Arm swinging is a typical feature of human walking: Continuous and rhythmic movement of the upper limbs is important to ensure postural stability and walking efficiency. However, several factors can interfere with arm swings, making walking more risky and unstable: These include aging, neurological diseases, hemiplegia, and other comorbidities that affect motor control and coordination. Objective assessment of arm swings during walking could play a role in preventing adverse consequences, allowing appropriate treatments and rehabilitation protocols to be activated for recovery and improvement. This paper presents a system for gait analysis based on Microsoft Azure Kinect DK sensor and its body-tracking algorithm: It allows noninvasive full-body tracking, thus enabling simultaneous analysis of different aspects of walking, including arm swing characteristics. Sixteen subjects with Parkinson’s disease and 13 healthy controls were recruited with the aim of evaluating differences in arm swing features and correlating them with traditional gait parameters. Preliminary results show significant differences between the two groups and a strong correlation between the parameters. The study thus highlights the ability of the proposed system to quantify arm swing features, thus offering a simple tool to provide a more comprehensive gait assessment.

## 1. Introduction

The rhythmic and symmetrical swinging of the arms is one of the primary pieces of evidence of healthy walking. It is so important that several studies have focused on why and how this pendulum-like movement occurs and its effects on walking [1]. The reason and origin of this oscillation are not yet entirely clarified [2]. For a long time, arm swing during walking was considered a merely passive phenomenon. Only later, a more in-depth analysis of the shoulder joints also suggested active muscle involvement, a hypothesis that was confirmed by studies using electromyography [3]. However, the debate on whether it is a purely passive, totally active, or only partially controlled phenomenon is still open [4,5]. Consequently, the analysis of arm oscillations during walking is a topic of great interest, especially in pathological conditions that may alter normal motor behavior [2].

According to some studies, arm swinging contributes to stability and optimization of energy expenditure during walking. The study by Ortega et al. [6] showed that arm swing helps balance the angular momentum of the body during walking, thus reducing the inherent lateral sway of the body’s center of mass and providing greater stability, whereas [7] investigated the effects of arm swing on local trunk stability during gait. In addition, a good arm swing contributes to better recovery after an external perturbation during walking, as demonstrated by [8]. Finally, a good arm swing minimizes energy consumption during walking, as demonstrated in [9]: In this study, the reduced arm swing was correlated with increased oxygen consumption and heart rate. All these findings are extremely relevant in diseases characterized by upper limb dysfunction since they have a direct impact on walking efficiency, stability, and fatigability of walking over long distances. Recently, increasing attention is being paid to arm movement during gait rehabilitation, especially in some neurological diseases such as Parkinson’s disease (PD), to improve gait patterns [10,11]. Several studies have shown that impaired arm movements during gait are common in PD [12,13]: Asymmetry, reduced amplitude and speed, altered rhythm and coordination, and a low number of arm swings are associated with clinical severity scores [14] and increased risk of fall [15], and are also a possible prodromal marker of the pathological condition since differences in arm swing have been detected between parkinsonian subjects and healthy controls [16,17]. Given the importance and implications of this aspect in walking patterns, interest in evaluating arm swing characteristics and effects has grown in recent years, moving from a primarily qualitative to a more quantitative analysis.

An in-depth analysis of the consequences of constrained arm swinging on healthy young adults was conducted in [18]. The main findings suggest that restriction of the arm swing, in one arm or both, worsens walking abilities in this population, as evidenced by a reduction in some relevant spatiotemporal parameters. A similar study was conducted in children with cerebral palsy [19], showing a reduction in walking speed associated with restricted arm movements under preferred and high-speed conditions. In [20], the effect of arm swing variation in unilateral trans-humeral amputees was evaluated, whereas [21] investigated the effects of restricted arm swing on the vertical displacement of the body’s center of mass as associated with excessive energy expenditure. In [22], the impact of age and gender on arm swing speed was analyzed, whereas [23] focused on the effects of arm swing on balance improvement in post-stroke subjects.

Regarding PD, the analysis of arm swing during walking mainly focuses on asymmetry and reduced amplitude with different purposes. As mentioned above, ref. [17] aimed to check whether changes in arm swing are related to genetic mutations as a prodromal marker of PD. Instead, ref. [16] focused on the analysis of arm swing alterations in early PD and its utility for differential diagnosis, ref. [24] investigated the improvements in arm swing due to dopaminergic medication and the changes associated with task complexity, and [25] investigated the relationship between arm swing asymmetry and gait parameters. Ref. [26] studied the effects of dopaminergic therapies on arm swing asymmetry and amplitude, ref. [27] used arm swing as an index of gait worsening under dual-task conditions, ref. [12] explored whether arm swing stimuli could improve walking patterns, and [11] had the same goal but using weights on arms.

Most of these studies used traditional instrumented gait-analysis systems (e.g., optoelectronic systems), which are the gold standard for motion capture in clinical settings due to their precision and accuracy [8,11,19,21,25,27,28].

On the other hand, several recent studies aimed to quantitatively measure arm swing features through cost-effective and minimally invasive devices that can be used in unsupervised environments or where traditional motion-capture systems are not applicable. Among them, refs. [29,30,31] used wearable sensors to compare arm swing features of PD subjects and healthy controls, ref. [32] used a smartphone to quantify arm swing and generate musical feedback to improve it, and [33] used ultrasound emitters to study the effects of age and mental load on arm swing.

Other approaches use contactless solutions to analyze gait patterns: They rely primarily on optical sensors and video analysis techniques to estimate walking features as traditional gait analysis. In particular, RGB-Depth sensors such as Microsoft Kinect^®^ (Microsoft Corporation, Redmond, WA, USA) have proven to be an alternative to wearable sensors in low-cost motion capture and analysis for clinical and rehabilitation purposes. These optical sensors have been used extensively for motion analysis [34,35,36,37] and rehabilitation goals [38,39,40] due to the availability of body-tracking algorithms capable of capturing body movements comprehensively, noninvasively, and in real time, generating a three-dimensional skeletal model from which to derive functional parameters for specific motor tasks, including gait analysis [41,42,43,44,45,46]. Recent developments in computer vision techniques and progress in computational resources (including processors and graphic cards) have led to new body-tracking methodologies based on neural networks to improve accuracy in human motion capture and pose estimation. This novel approach was used, for example, in [47,48,49] for gait analysis and fall detection, with the limitation of being a 2D approach that prevents 3D measurements. However, the same methodology has been integrated into the body-tracking algorithm of the third generation of Kinect sensors (i.e., Microsoft Azure Kinect DK), enabling 3D motion capture due to distance (i.e., depth) estimation provided by the optical sensor. Several studies have verified and validated the performance of the new device compared to its predecessors [50], demonstrating its higher accuracy in terms of on-board sensor and body tracking [51,52] and its suitability for motion analysis [53,54,55,56] even compared to gold reference systems. 

However, only a few studies have analyzed arm swing during gait using optical approaches [57,58,59,60]. However, to our knowledge, none have exploited the potential of the new Azure Kinect and its improved 3D body-tracking algorithm in this context, which is particularly important, for the reasons mentioned above, for the early detection of any alterations that could affect walking safety.

To fill this gap, in this study, we propose integrating arm swing into gait analysis for a more comprehensive evaluation of walking patterns, using a motion-capture system based on the new Azure Kinect, as in [56]. Indeed, the potential for body tracking provided by the optical sensor makes it possible to capture the movement of all body segments simultaneously and to evaluate different aspects of gait accordingly, unlike, for example, wearable sensors limited to the segments on which they are placed. Using the same approach as in other studies [41,46,55], the system is able to estimate a subset of spatiotemporal parameters, as in traditional instrumented gait analysis, and parameters related to center-of-mass (COM) sway during gait that explains the main features of walking patterns. Possible parameters related to arm swing can contribute to the complete and comprehensive characterization of walking. For this purpose, the proposed system and methodology were applied to groups of healthy subjects and subjects with PD.

The main objectives of this study include verifying the system’s ability to measure arm swing-related parameters during walking and detecting differences between the two groups of subjects, as well as for spatiotemporal and COM-related parameters; defining objective indicators of the asymmetry and synchrony of arm swing movements that could easily detect transition to pathological gait over time; and verifying the correlation between arm swing parameters and others that characterize walking pattern (i.e., spatiotemporal and COM-related parameters). In this phase, the present study does not aim to assess the correlation between parameter changes and disease severity.

Preliminary results suggest that the system allows for comprehensive and quantitative assessment of walking strategies and characteristics.

## 2. Materials and Methods

### 2.1. Kinect-Based Motion-Capture System

The motion-capture system used in this study includes three elements: a processing unit (which can be a mini-PC or a laptop), a single RGB-Depth camera (i.e., the Microsoft Azure Kinect DK [61]), and a monitor (or TV screen) for displaying the system’s graphical user interface (GUI) and providing visual feedback of body movements for interaction with the system. The optical device provides synchronized color, depth, and infrared video streams with a maximum frame rate of 30 fps (frames per second). Other parameters are configurable during device initialization: For this study, we set a 1080p resolution for color stream and narrow field of view (NFW) for depth stream to ensure accurate body tracking at a distance compatible with the needs of gait analysis. Two native software development kits (SDKs) provide access to the device and body-tracking capabilities. Specifically, the body-tracking algorithm uses a deep-learning approach based on neural networks to reconstruct a 3D skeletal model (32 virtual joints) that maps human body movements in real time [62]. This approach is expensive in terms of computational resources, so the processing unit was chosen with hardware features that meet the requirements for real-time body-tracking operations. Specifically, the following hardware was used for this study: mini-pc ZOTAC© (Zotac, Fo Tan, New Territories, Hong Kong, China) ZBOX EN52060-V model, 9th generation Intel^®^ Core^TM^ processor (2.4 GHz quad-core), 16 GB of RAM, NVIDIA GeForce RTX 2060 6GB GDDR6. The mini-PC is equipped with an HDMI port to connect an external monitor and some USB3 ports for connecting the Azure Kinect sensor. The system has Windows 10 as its operating system.

**Figure 1:**
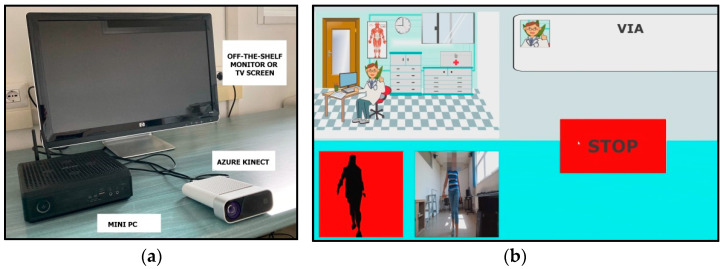
(**a**) The proposed system with the mini-PC, Azure Kinect, and a monitor; (**b**) GUI for the acquisition of gait trials.

In addition, custom software in C#, developed in Unity^®^ (Unity Technologies, San Francisco, CA, USA), runs on the system, both to manage the acquisition procedure (i.e., the process of collecting and saving data on the skeletal model) and to implement the human–machine interaction and related GUI. The system saves information about each joint in the skeletal model (including positions, rotations, and confidence) in JSON format files for the next step, which analyzes 3D trajectories to estimate gait features. The user interface, suitable even for people with low technological skills, makes the system easy and intuitive to use: Each GUI includes a few interactive buttons (to manage data acquisition and saving procedures) and audio and text messages that guide the patient while performing the gait task. The layout of the GUI (including object location, font size, and colors) and the size of the monitor (at least 26 inches) make the system suitable in case of sight problems typical of the elderly population. The proposed system and an example of the GUI are shown in Figure 1a and Figure 1b, respectively.

### 2.2. Recruitment Procedure and Experimental Protocol

For this preliminary study, approved by the internal ethical committee of Istituto Auxologico Italiano, we recruited 16 subjects with PD among patients in the Division of Neurology and Neurorehabilitation at San Giuseppe Hospital, Istituto Auxologico Italiano, Piancavallo (Verbania), Italy. The recruitment procedure established inclusion and exclusion criteria. General inclusion criteria included the ability to walk 10 meters without aids (the use of canes or other supportive tools, typical in more impaired subjects, was avoided because it interferes with the assessment of arm swing) and to understand the instructions provided by the system and supervisor during the experimental session. Specific inclusion criteria for PD subjects are related to tremor severity (≤1, according to the Unified Parkinson’s Disease Rating Scale [63]) and the Hohen and Yahr (H&Y) score in the range of 1–3. These constraints were also set to identify potential subjects with PD who could benefit from remote monitoring in the home environment. General exclusion criteria included previous neurosurgical interventions, neurological and musculoskeletal disorders due to other diseases, cognitive disorders assessed by Mini-Mental State Examination (MMSE < 27/30), and cervical–dorsal or shoulder–upper limb comorbidities that could affect the outcome of the analysis. In contrast, no criteria related to age, sex, therapy, or side dominance were included in the recruitment procedure.

For the study objectives, we also recruited 13 healthy control subjects among the caregivers and relatives of the subjects with PD. The same general inclusion and exclusion criteria were applied to the healthy subjects, except, of course, the specific inclusion criteria for subjects with PD.

All selected participants signed the informed consent form after a detailed explanation of the experimental procedure and before the start of the data acquisition campaign, which was conducted in accordance with the Institute’s ethical standards, the Declaration of Helsinki, and its amendments.

The experimental setup was the same as in our previous studies [41,46] to ensure the accuracy of the body-tracking algorithm within a virtually defined gait analysis path (GAP) on a traditional 10-meter walkway. The experimental campaign took place in a hospital setting, under the supervision of clinical and technical staff who managed data acquisition using the proposed system according to the experimental protocol: Both groups performed the test session under the same conditions.

According to the experimental protocol, the test session consisted of two phases. In the first, each subject was instructed by the supervisor to perform three walking trials along the 10-meter walkway to become familiar with and understand the acquisition procedure. No data were collected at this stage. In contrast, data were collected during the second phase, in which the subject was asked to maintain an upright posture for a few seconds before walking, at a normal pace, toward the optical device (i.e., Azure Kinect) placed at the end of the walkway: The subject, in this way, entered the GAP at maximum walking ability. Each participant performed three repetitions of walking at this stage, with a 1-minute break between each. At the end of each walking test, the system saved data in JSON format files for further processing and analysis.

### 2.3. Data Analysis

Data analysis relies on offline processing of 3D joint trajectories acquired during gait tests and saved in JSON files, and the analysis procedure includes two stages. The first stage involves preprocessing the collected data using resampling and filtering techniques. Since the variable framerate (about 30 fps) of the optical sensor introduces jitter into the timestamp of the data, 50 Hz resampling (cubic interpolation) is used to obtain a uniform time baseline: Up-sampling of the data was preferred to increase the density of the samples and better refine the 3D trajectories. Then, a low-pass filter (8 Hz) was applied to the resampled data to remove high-frequency noise: The cutoff threshold allows movements to be captured during gait in healthy and pathological subjects [64,65]. The second stage consists of custom MATLAB scripts that work on preprocessed trajectories and analyze the three main aspects of gait: spatiotemporal, body sway, and arm swing features.

Regarding spatiotemporal and body sway features, the analysis procedure was the same as described in [41,46]. Precisely, a subset of traditional spatiotemporal parameters was estimated through the step segmentation algorithm that works on the 3D trajectories of the left (ANK_L_) and right (ANK_R_) ankle joints of the skeletal model to analyze the gait performance within the GAP. The analysis of the body sway relies on the 3D trajectories of the left (HIP_L_) and right (HIP_R_) hip joints of the skeletal model from which to calculate the 3D segment midpoint (COM_HIP_) and evaluate body sway along the mediolateral and vertical directions during walking.

Arm swing analysis consists of linear and angular measurements of arm movements within the GAP zone and aims primarily to quantify arm swing amplitude (or magnitude). Linear measures are calculated by separately considering the x, y, and z components of the left wrist (WRIST_L_) and right wrist (WRIST_R_) joints of the skeletal model: This information is used to estimate the displacement of the wrists relative to the pelvis (PELVIS) joint of the skeletal model along the mediolateral, anteroposterior, and up–down directions [16,27,59]. Angular measures are calculated by considering the left arm (SHOULDER_L_–WRIST_L_) and right arm (SHOULDER_R_–WRIST_R_) segments of the skeletal model: This information is used to estimate the relative angle between the arm segments and the vertical segment of the same side, which are the (SHOULDER_L_–HIP_L_) and (SHOULDER_R_–HIP_R_) segments, respectively [11,17]. Linear and angular measures are also estimated separately for the anterior (i.e., forward) and posterior (i.e., backward) phases of the full swing motion in order to provide a more in-depth analysis [11,24,59].

Another relevant feature of arm swing is asymmetry. Several methods are commonly used to quantify asymmetry in the arm swing, but one of the most applied is the symmetry angle (SA) proposed in [66]: This index expresses the relationship between the discrete measurements relative to the left and right sides of the body. Zero values indicate perfect symmetry; increasing values indicate increasing asymmetry. In this study, we used an absolute measure of symmetry angle as in [11,16,17,26,59] and defined as in Equation (1):

ASA = abs ((45° − arctan (P_MORE_/P_LESS_))/90°) × 100 (%), (1)

where P_MORE_ and P_LESS_ are the parameter values associated with major and minor arm swing, respectively. As mentioned above, both linear and angular measurements of arm swing amplitude are estimated and, therefore, the ASA index was calculated for some representative parameters.

Finally, we included another potentially relevant feature: the synchrony index (SI) between the trajectories of the left and right arms (arm–arm) and between the trajectories of the arm and opposite leg (arm–leg) during walking, which could provide further information about the walking pattern. In fact, we expected there to be a relationship between the arm–arm and arm–leg movements, and that the lack of such synchrony may indicate a gait disturbance. For this assessment, we used a simple Pearson correlation between the arm segment (SHOULDER–WRIST) and the opposite leg segment (HIP–ANKLE): Higher correlation coefficients indicate greater synchrony between limb movements and, probably, a better and more efficient walking pattern.

We calculated the same synchrony index between left and right arm trajectories, specifically considering only the z-component (i.e., anteroposterior direction), which is the most significant during walking. We also expected there to be a relationship between the movements of the two arms (in particular, a negative relationship, since they move in opposition): Higher correlation coefficients (as absolute values) should indicate greater synchrony between arm movements and, probably, a better and more efficient walking pattern. If this proves to be true, SI could become an indicator for detecting the transition from healthy to impaired gait.

The next section describes the estimated parameters for gait characterization according to the three aspects that have been mentioned: arm swing, spatiotemporal features, and sway of the body center of mass.

### 2.4. Characterization of Gait through Objective Parameters

From the 3D joint trajectories and body segments collected during the walking trials, the analysis procedure estimated specific parameters related to arm swing, center-of-mass sway, and walking pattern (i.e., spatiotemporal parameters). The following tables identify the estimated parameters for each aspect: They were computed for each walking trial of the experimental campaign. Table 1 shows the arm swing linear and angular measurements. 

The SWAY_ANT parameters were estimated from the WRIST_L_ and WRIST_R_ trajectories and represent the maximum anterior sway relative to the PELVIS joint. Similarly, the SWAY_POS parameters represent the maximum backward sway relative to the PELVIS joint. These parameters were estimated separately along the three directions of motion (anteroposterior, mediolateral, and up–down). The SWAY_RANGE parameters represent the maximum amplitude of full-arm swing, i.e., the peak-to-peak distance between maximum anterior and maximum posterior swing. The PATH_TOT_ parameter is the total 3D distance traveled by the wrist within the GAP zone, whereas the SWAY_AREA is the area determined by the convex hull enclosing the wrist motion along the AP and ML directions. Considering that the motion along the AP direction probably is the most representative of the arm swing, only the maximum velocity along the AP direction was considered (SPEED_AP). The ANGLE_ANT parameters were estimated, for the left and right arms, from the SHOULDER–WRIST and SHOULDER–HIP segments and represent the maximum anterior angle between them. Similarly, the ANGLE_POS parameters represent the maximum posterior angle between the two segments. The ANGLE_RANGE parameters are the maximum angle range, i.e., the peak-to-peak distance between the maximum anterior angle and the maximum posterior angle. The ASA parameters were calculated from Equation (1) for the maximum anterior angle (ASA_ANGLE_), the path traveled (ASA_PATH_), and the maximum anterior sway along the AP direction (ASA_AP-RANGE_): The ASA values were obtained by substituting the generic P_MORE_ and P_LESS_ elements of the equation with the largest and the smallest values of the considered parameter, respectively. Instead, Table 2 shows the subset of parameters relative to gait analysis, as in [41,46].

As in traditional gait analysis, some parameters were estimated separately for the left and right sides of the body; other parameters, however, were calculated as representative of the entire gait. All the spatiotemporal parameters were estimated from the ANKLE_L_ and ANKLE_R_ trajectories within the GAP zone through the step segmentation algorithm using the z-component of the 3D joint positions exclusively: the z-component reflects the motion in the anteroposterior direction toward the optical device.

The STEP_LEN_, STEP_WIDTH_, STEP_VEL_, and STEP_TIME_ parameters were averaged over the number of left and right steps, respectively, detected within the GAP zone. The same applies to the gait cycle parameters (i.e., STRIDE_LEN_, DOUBLE_SUPP_, STANCE_DUR_), which were averaged over the number of full left and right strides detected within the GAP zone. STEP_NUM_ and STRIDE_NUM_ are the total numbers of steps and complete gait cycles (i.e., the sum of left and right sides) detected within the GAP zone. GAIT_VEL_ is a more general parameter that characterizes gait and represents overall speed calculated as the ratio of the length of the GAP zone to the time taken to travel through it (i.e., from the time the body enters the GAP to the time it leaves the GAP). CADENCE is also an overall gait parameter and represents an estimation of the number of steps in a minute.

The ML_SWAY_ and V_SWAY_ parameters were estimated from the trajectory of COM_HIP_ within the GAP zone and represent the maximum absolute lateral and vertical oscillations from the straight walking to the optical sensor.

### 2.5. Statistical Analysis

The arm swing, center of mass, and spatiotemporal parameters were estimated for two of the three walking tests collected on all participants.

Due to the small size of the collected dataset, the Shapiro–Wilk test was used to check the distribution normality of each estimated parameter. Thus, the statistical analysis included parametric or non-parametric tests depending on the individual case. Since the Shapiro–Wilk normality test showed a non-normal distribution for all parameters, we continued the statistical analysis using mainly nonparametric tests. Notably, all the parameters in Table 1 deviated significantly from the normality hypothesis: many of them with *p* < 0.001, some with *p* < 0.01 (i.e., SWAY_ANT_UD_, SWAY_RANGE_ML_, SPEED_AP_, ANGLE_POS), and others with *p* < 0.05 (i.e., SWAY_ANT_AP_, SWAY_POS_AP_, SWAY_RANGE_AP_, ANGLE_ANT, ANGLE_RANGE). Regarding Table 2, almost all parameters deviated significantly from the normal distribution hypothesis with *p* < 0.001, except for STEP_LEN_, STEP_VEL_, STRIDE_LEN_, GAIT_VEL_, and V_SWAY_, which showed *p* < 0.01.

Next, the estimated parameters for the two groups were compared through the nonparametric Mann–Whitney U test for independent samples to detect a statistical difference between them: The median values with related first and third quartiles were calculated for each parameter. However, considering that the two groups were borderline to be considered large samples, we also used the Student’s *t*-test (parametric) to support the statistical significance analysis of the parameters.

Spearman’s rank correlation (also a nonparametric test) was used to study the correlation existing between the three categories of parameters (arm swing, spatiotemporal, and center of mass). The analysis was performed separately for the control group and the PD group to see whether there were similarities or differences in the relationship between the parameter categories for the two groups. However, considering that the two groups were borderline to be considered large samples, we also used the Pearson’ correlation coefficient (parametric) to support the analysis of correlation between parameter pairs.

Statistical tests were performed using Jamovi (version 2.2.5), an open-source modular platform for statistical computing [67]: A 95% significance level (*p* < 0.05) was considered, where applicable, for the statistical tests.

## 3. Results

### 3.1. Participants Features and Data Collection

The PD subjects recruited for the study had the following demographic and clinical characteristics: 68.9 ± 7.1 years (average age), 8.9 ± 7.6 years (average disease years), 34.0 ± 6.5 pts (average UPDRS score), 2.1 ± 0.9 pts (average H&Y score), 164.5 ± 7.4 cm (average height), 77.2 ± 14.1 kg (average weight), and 5 females and 11 males (gender). The healthy controls recruited for this study had the following demographic characteristics: 56.3 ± 8.7 years (average age), 167.8 ± 7.4 cm (average height), 66.2 ± 10.9 kg (average weight), and 5 females and 8 males (gender). Although the two groups were not strictly homogeneous, this fact did not affect the objectives of the study.

Almost all the participants were able to perform all three walking trials as required by the experimental protocol. No trial was discarded due to technical or environmental issues during the acquisition phase.

### 3.2. Statistical Analysis on Estimated Parameters

The statistical analysis relied on two of the three walking trials performed by each subject. In fact, because the first trial of some subjects with PD did not fully comply with the protocol directives (e.g., subjects did not walk at their normal pace, or partially turned toward the supervisor for clarifications, or halted too early), we decided to include in the statistical analysis only the last two trials of each subject that, in contrast, were performed correctly. Therefore, the dataset available for the statistical analysis includes 32 walking trials for the PD group and 26 walking trials for the healthy controls. This means that for the parameters estimated separately (i.e., for the left and right sides of the body), 64 and 52 samples, respectively, were available for statistical analysis, whereas for the parameters estimated over the entire gait, 32 and 26 samples, respectively, were available.

As mentioned above, the Mann–Whitney U test for independent samples was used to test the statistical differences between the two groups and the discriminative power of each parameter. Results of Student’s *t*-test were also reported to support nonparametric analysis. Table 3 shows the results for the arm swing parameters and Table 4 for the spatiotemporal and center-of-mass parameters: The tables report the median and quartiles (first and third) for each group, along with the rank-sum test statistic, *p*-value, and significance level.

The results suggest that only some of the estimated arm swing parameters significantly differed between the two groups of subjects. The interquartile ranges indicated a partial overlap for parameters showing a statistically significant difference between the two groups. In contrast, parameters that were not statistically significant showed almost complete overlap between HC and PD groups. The Student’s *t*-test was in agreement with the nonparametric test except for the SWAY_AREA and SWAY_RANGE_UD_ parameters, whose significance level was slightly lower. The main significant parameters were those related to anterior arm motion, as linear (SWAY_ANT) and angular (ANGLE_ANT) measures. In contrast, the parameters related to backward arm motion (SWAY_POS and ANGLE_POS) did not appear to be relevant. As expected, the anteroposterior (and its SPEED_AP_ velocity) and up–down directions better characterized the arm swing motion, whereas the mediolateral component was similar in the two groups. The amplitude parameters (SWAY_RANGE and ANGLE_RANGE) also denoted a significant difference between the two groups, except for the amplitude in the mediolateral direction. The SWAY_AREA parameter, which simultaneously takes into account the anteroposterior and mediolateral directions, was also significant, although to a lesser degree. This result probably depends on the inclusion of mediolateral direction leading to more similar area values. This behavior was also reflected on the PATH_TOT_ parameter, which was instead calculated by considering all three directions. In any case, all the parameters detected a significant difference between the two groups for the defined significance level (*p* < 0.05).

The interquartile ranges in Table 4 indicate a partial overlap for parameters showing a statistically significant difference between the two groups. In contrast, parameters that were not statistically significant showed almost complete overlap between HC and PD groups. The Student’s *t*-test was in agreement with the nonparametric test except for the STEP_WIDTH_, which was not significant, and for CADENCE and DOUBLE_SUPP_ parameters, whose significance level was slightly higher. The results in Table 4 also suggest that all the estimated spatiotemporal parameters showed a significant difference between the two groups of subjects, except for CADENCE. The parameters related to the center of mass (COM_HIP_) also did not significantly differ between the two groups. This result may be because lateral and vertical sways during walking are not necessarily present in PD subjects (body sway is a feature that mainly occurs in more advanced stages of the disease). These parameters were included because previous studies have shown that lateral sway may highlight gait abnormalities in pathological subjects [41,46]. An in-depth analysis of body sway over all participants revealed a difference in mean, standard deviation, minimum, and maximum values for the lateral sway for the two groups. In fact, for ML_SWAY_, it was obtained (mean: 56.72 ± 13.52 mm; min: 37.67 mm; max: 96.10 mm) for the HC group versus (mean: 63.15 ± 26.97 mm; min: 29.44 mm; max: 164.44 mm) for the PD group. The standard deviation showed greater variability in the lateral body sway (ML_SWAY_) for the PD group, which included subjects with very pronounced lateral body sway (as evidenced by the maximum value). It follows that body sway analysis is also important for identifying gait abnormalities.

### 3.3. Asymmetry and Synchrony Indices

The same statistical analysis was performed on asymmetry coefficients and synchrony indices. Table 5 reports the obtained results.

The results confirm that the asymmetry was more pronounced in the PD group, with a statistically significant difference for all three parameters. In this case, the interquartile range also indicated only partial overlap for parameters showing a statistically significant difference between the two groups. In contrast, parameters that were not statistically significant showed almost complete overlap between the HC and PD groups. The Student’s *t*-test was in agreement with the nonparametric test except for the SI_ARM-LEG_ parameter, whose significance level was slightly higher. The ASA_PATH_ index was the least significant, but this result agreed with the lower significance of the PATH_TOT_ parameter. The same is true for the ASA_ANGLE_ and ASA_AP-RANGE_ indices associated with ANGLE_RANGE and SWAY_RANGE_AP_ parameters, which showed higher significance. It is important to note a similarity between the magnitudes of the three asymmetry parameters in both the PD and HC groups, meaning that the same level of asymmetry was consistently detected across both linear and angular measures.

Both synchrony indices, measured through Pearson’s correlation coefficient, showed high values, which means that there was, on average, a strong correlation between arm swing and opposite leg movements (SI_ARM-LEG_) and opposition movements of the two arms (SI_ARMS_). The SI_ARM-LEG_ index takes positive values, as the movement of the opposite arm and leg occurs in the same direction. In contrast, the SI_ARMS_ takes negative values, as the trajectories of the arms are in opposition. In any case, absolute coefficients greater than 0.7 indicate a strong correlation between the trajectories and, consequently, high synchrony (i.e., coordination) of movements during walking. Statistical analysis of SI_ARMS_ indicated a significant difference between the PD and HC groups: Interestingly, the quartile values showed more significant variability for the PD group than the HC group. In contrast, the SI_ARM-LEG_ seemed less significant: In this case, the parameter range was wider, especially on the left side (corresponding to the lowest correlation values), as suggested by the first quartile. 

The presented results suggest that these indices (high values for ASA and low values for SI) could be effectively used to quantify specific gait abnormalities associated with asymmetrical walking patterns and limited coordination.

### 3.4. Arm Swing in PD and Healthy Control Groups

**Figure 2:**
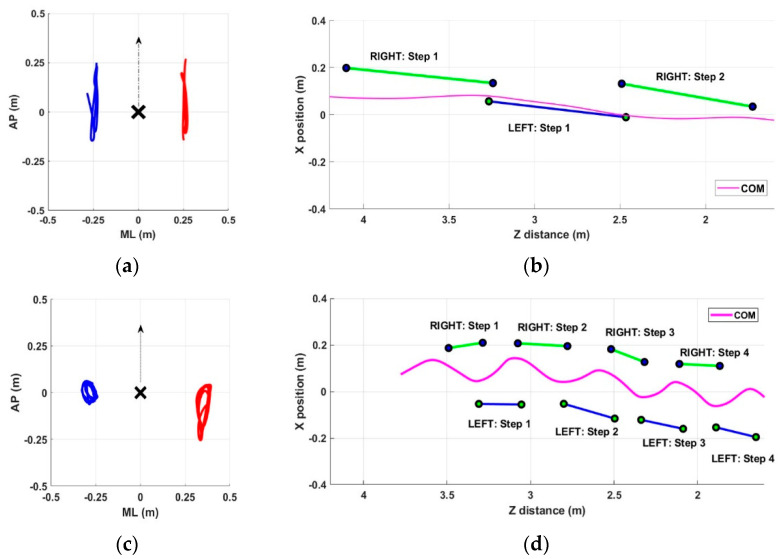
Example of arm swing trajectories (in meters) and gait patterns for PD subjects. (**a**) Subject #10: arm swing in AP and ML directions for left arm (red line) and right arm (blue line) relative to pelvis joint (black cross); (**b**) subject #10: gait patterns with detected steps and center-of-mass trajectory within the GAP zone; (**c**) subject #6: arm swing on AP and ML directions for left arm (red line) and right arm (blue line) relative to pelvis joint (black cross); (**d**) subject #6: gait patterns with detected steps and center-of-mass trajectory within the GAP zone.

**Figure 3:**
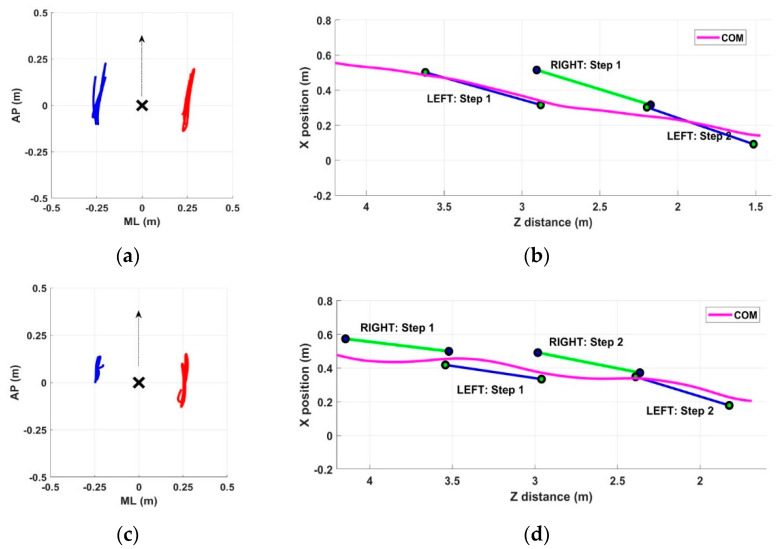
Example of arm swing trajectories (in meters) and gait patterns for HC subjects. (**a**) Subject #5: arm swing in AP and ML directions for left arm (red line) and right arm (blue line) relative to pelvis joint (black cross); (**b**) subject #5: gait patterns with detected steps and center-of-mass trajectory within the GAP zone; (**c**) subject #3: arm swing in AP and ML directions for left arm (red line) and right arm (blue line) relative to pelvis joint (black cross); (**d**) subject #3: gait patterns with detected steps and center-of-mass trajectory within the GAP zone.

In this section, we present some examples of arm swing and gait patterns referring to both healthy and pathological subjects. The intent is to show the ability of the proposed system to detect all aspects of gait performance qualitatively. The graphical representation allows for a quick and intuitive qualitative overview of any gait problems that can then be measured objectively through quantitative parameters. In particular, Figure 2 shows examples referring to subjects in the PD group, whereas Figure 3 shows examples referring to subjects in the HC group. For each group, two significant subjects were considered: a PD subject with normal gait (Subject #10), a PD subject with altered gait (Subject #6), an HC subject without gait problems (Subject #5), and an HC subject with asymmetry in arm swing (Subject #3). These figures also aim to show that gait abnormalities were not necessarily present in all recruited PD subjects and that, on the contrary, some healthy subjects may have had some slight abnormalities. In both cases, a quantitative assessment could allow for early correction of the problem and improve the overall quality of gait.

As shown in Figure 2 for the PD group, subject #10 showed symmetry in arm swing (Figure 2a) and a fast and stable gait characterized by long steps (Figure 2b). In contrast, subject #6 showed significant asymmetry in the arm swing, with the right arm having almost no movement (Figure 2c) and a gait characterized by short strides and lateral oscillations of the center of mass (Figure 2d).

Regarding the healthy control group, subject #5 showed symmetry in arm swing (Figure 3a) and a fast and stable gait characterized by long steps (Figure 3b). In contrast, subject #3 showed slight asymmetry in arm swing (Figure 3c) and a gait characterized by shorter steps and slight lateral center-of-mass sways (Figure 3d).

The qualitative assessment obtained from the graphs is supported quantitatively by the previously described parameters. Table 6 and Table 7 show the values of the most significant parameters (according to statistical analysis), estimated for the four subjects.

As previously described, the subjects selected for this comparison represent borderline and significant cases for both groups. In any case, the objective measures confirmed the initial indications provided by the graphic representations. In particular, the estimated parameters for PD subjects agreed with the assigned H&Y score, with 1 for subject #10 and 3 for subject #6.

Some relevant insights emerged from the comparison. Subject #6 (PD group) showed alterations in all gait characteristics (arm swing, lateral sway, spatiotemporal parameters). Regarding arm swing, all ASA parameters showed rather great values, whereas the SI_ARMS_ had the lowest value compared to the other cases (this is consistent with restricted movement of the right arm). This evidence could thus confirm the use of the SI_ARMS_ as an indicator of alterations in arm movement synchrony. Subject #10 (PD group) showed comparable performance to subject #5 (HC group) for all gait characteristics, including the SI_ARMS_: This subject appeared to have a motor pattern similar to healthy subjects (this is consistent with his early stage of the disease). In contrast, subject #3 (HC group) showed arm swing asymmetry in terms of amplitude but not asynchrony, considering that the SI_ARMS_ index showed a very high value.

### 3.5. Correlation between Type of Parameters

**Figure 4:**
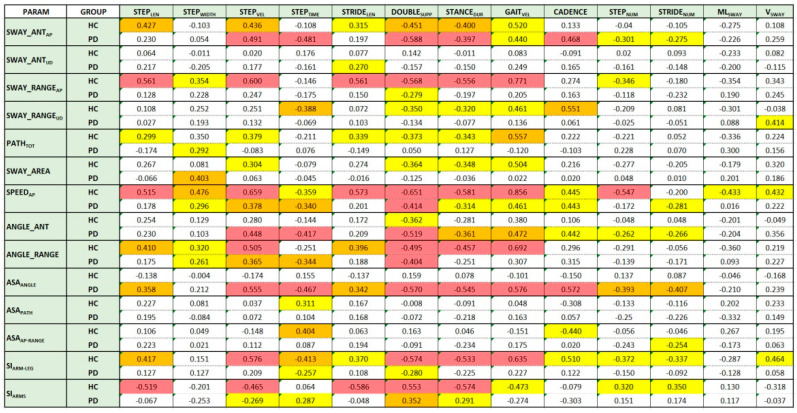
Spearman’s correlation coefficients between parameters for HC and PD groups. Colored boxes indicate the level of significance: yellow (*p* < 0.05); orange (*p* < 0.01); red (*p* < 0.001).

The last analysis aimed to test the relationships between the estimated parameters of the three categories: arm swing, spatiotemporal, and center of mass. Spearman’s correlation coefficient was used to measure the existing correlation. Positive values indicate a direct relationship (i.e., the parameter pair moves in the same direction); negative values indicate an inverse relationship (i.e., the parameter pair moves in the opposite direction). Larger values indicate strong correlations; smaller values indicate weak correlations. Figure 4 shows the Spearman’s correlation values for the HC and PD groups. The background color of the boxes indicates the significance level (*p*-value) of the Spearman’s correlation coefficient (red: *p* < 0.001; orange: *p* < 0.01; yellow: *p* < 0.05). White boxes refer to parameter pairs without significant correlation. The same analysis performed using Pearson’s correlation coefficient confirms the behavior shown in Figure 4: The differences in terms of correlation were in fact negligible and did not change the corresponding level of significance.

The analysis was performed separately on the two groups to reveal any differences in correlation between different walking aspects. The distribution of the colored boxes (parameter pairs with significant correlations) shows differences between the two groups at first glance.

For the HC group, there seemed to be an overall more significant correlation than for the PD group between arm swing and spatiotemporal parameters. This is evident both by looking at the distribution of yellow boxes (there are many more for the HC group) and by looking at the magnitude and significance of the relationship (in general, larger and more significant coefficients). This result seems to support the hypothesis of the importance of arm movement for effective and healthy walking. For the HC group, it seems that the arm swing parameters most correlated with spatiotemporal parameters were the linear parameters related to anteroposterior motion (SWAY_ANT_AP_, SWAY_RANGE_AP_, SPEED_AP_), whereas for the PD group, angular parameters seemed to be more significant (ANGLE_ANT, ANGLE_RANGE).

Regarding the asymmetry indices, none seemed to correlate with the spatiotemporal parameters for the HC group. This may be because arm swing asymmetry was a sporadic condition in healthy subjects. In contrast, for the PD group, the ASA_ANGLE_ parameter showed a significant correlation with all spatiotemporal parameters except STEP_WIDTH_.

Regarding the synchrony indices, practically the opposite happened. The indices for the HC group (SI_ARMS_ and SI_ARM-LEG_) showed a good correlation with almost all spatiotemporal parameters (especially the most significant ones). In contrast, neither index showed an equally good correlation for the PD group. This result suggests that good synchronism, both in arms and opposite arm–leg movement, could provide an indication of a gait without particular alterations.

Finally, for both groups, none of the arm swing parameters seemed to significantly correlate with the parameters related to center-of-mass sways (ML_SWAY_ and V_SWAY_) for both groups. This result would seem to suggest that the anomalies associated with center-of-mass sway represent a separate category, unrelated to the other types of gait anomalies.

## 4. Discussion

Arm swing is a typical feature of the human gait. Numerous studies have proven that it ensures effective and stable walking, and responsiveness in recovering balance following external perturbations. Reduced arm swing can therefore impact the overall quality of walking. Several factors can affect this movement, which can therefore be considered a walking disorder. In addition to accidents and injuries that can temporarily prevent good arm swings during walking, other chronic conditions can permanently alter arm swings—for example, diseases characterized by symptoms that impair movement, such as Parkinson’s disease (PD). Recently, the importance of effective arm swing in PD has been recognized, and treatment of its alterations has become part of several gait rehabilitation protocols with the aim of improving gait patterns [10,11]. Typical alterations of arm swing in PD include asymmetry, reduced amplitude and speed, and asynchrony of movement, which occur mainly and more frequently with progression and increasing disease severity [14].

In the last few years, there has been a shift from qualitative to quantitative assessment of arm swing and its characteristics, considering the importance of this gait aspect. In some cases, assessment of arm swing has been included in traditional gait analysis using optoelectronic systems, the gold standard for motion analysis, but the complexity and the need to place markers on the body limit assessment with these systems in clinical settings. Instead, it would be helpful to have inexpensive and minimally invasive assessment systems suitable for unsupervised settings. These systems could allow for frequent monitoring of gait as a whole and early detection of changes in arm swing movements so that specific rehabilitation protocols could be promptly activated and the risk of more severe consequences (e.g., falls) reduced, especially in frail and pathological individuals.

Along this line of research, this study presents a noninvasive motion-capture system based on the new Azure Kinect [56] to obtain a quantitative and combined assessment of different aspects of gait, including traditional spatiotemporal parameters, center-of-mass sways, and arm swings. We consider that these three aspects, taken individually or together, could provide relevant insights into the overall quality and gait evolutions in parkinsonian people compared to “healthy” gait, with the advantage of having an easy and portable tool to measure them.

The main objectives of the study are summarized here. The first objective was to test the ability of the system to measure gait features and detect statistically significant differences between parkinsonian and healthy gait patterns in arm swing, spatiotemporal, and center-of-mass parameters. The second objective was to define indices to quantify asymmetry and synchrony related to arm swing that can be monitored over time to detect the onset of gait disturbances, perhaps before spatiotemporal and stability features. The last objective was to verify the relationship between parameters related to the three aspects of gait analysis in healthy and parkinsonian subjects. This analysis could help detect the onset of walking abnormalities, revealing the transition from normal to pathological gait. In the future, the present study will be extended to investigate the correlation between parameter changes and PD severity.

Regarding the first objective, the results demonstrate the system’s ability to qualitatively detect differences in different aspects of walking (Figure 2 and Figure 3), which are also reflected in objective measures through specific parameters (Table 6 and Table 7). The parameters related to arm swing were defined based on the primary studies in the literature focused on arm swing, both in terms of the range of motion [11,16,17,24,27,59] and asymmetry [11,16,17,26,59]. In addition, we considered a subset of traditional spatiotemporal parameters and measures related to center-of-mass sway, as in our previous studies [41,46], that proved relevant in pathological conditions. Finally, we defined some indices of synchrony that aimed to quantify the coordination and synchronism of the movement of opposite arms and legs.

Regarding arm swing, the statistical analysis indicates that only a few parameters significantly differed between the two groups. In particular, the most significant were associated with the anterior phase of the arm swing rather than the posterior one. Regarding asymmetry, statistical analysis suggests the lower significance of ASA_PATH_ (*p* < 0.05) compared to ASA_ANGLE_ and ASA_AP-RANGE_ (*p* < 0.001). This is consistent with the lower significance of the PATH_TOT_ parameter (*p* < 0.05). About synchrony, both indices detected statistically significant differences, but particularly SI_ARMS_ (*p* < 0.001), which quantifies synchronism (i.e., coordination) between opposite arm movements. Difficulty in arm movement coordination could therefore be indicative of the onset of a subsequent gait disorder. All spatiotemporal parameters showed a significant difference between the two groups, except cadence, whose mean value for the PD group (104.99 steps/min) is still comparable to other studies focusing on gait analysis [68,69]. In contrast, center-of-mass parameters did not indicate a statistical difference between the two groups. However, the analysis of the mean and standard deviation, and minimum and maximum values, revealed more significant variability in the PD group and the presence of subjects with high lateral sway; this suggests that, although not relevant for discriminating between the two groups, analysis of center-of-mass motion is essential for a complete gait assessment.

Compared with other studies, our results agree with those of [16], where higher asymmetry and lower gait velocity were measured for PD subjects. In addition, no significant difference was found between “more” and “less” arm swing parameters of the PD and HC groups. This is in line with our PATH_TOT_ parameter (which has lower significance) but not with the sway range in specific directions (AP and UD), which, on the contrary, showed a statistical difference between the two groups (*p* < 0.001). This result could be due to the different composition of the PD group and the inclusion of subjects with greater severity. Our results regarding arm swing amplitude and asymmetry are also consistent with those reported in [59], which showed a significant reduction in arm swing amplitude and speed, and a significant increase in ASA for the PD group compared to healthy controls. The mean values of the angular measures of the arm swing were much higher than in [11], both considering the single phases (anterior and posterior) and the whole angular range. This is probably due to the different composition of our PD group, which also included less severe subjects compared with [11], where the H&Y score was between 2 and3 (our study also involved subjects with H&Y < 2). The same is true for spatiotemporal parameters that showed higher values and range than in [11]. Compared with [25], who reported results on arm swing and spatiotemporal parameters, our results are aligned with the significance of arm swing amplitude and angular asymmetry but not with the significance of spatiotemporal parameters, which were statistically significant in our study. Again, this is probably due to the inclusion of more severe subjects in our PD group compared with [25], where the mean H&Y score was 2.05 ± 0.29 (in our study, the mean H&Y score was 2.10 ± 0.9, which confirmed the inclusion of more severe subjects). This may have increased the differences with the healthy group.

Compared with [14,26], our results on asymmetry are also consistent: In both studies, the PD group showed reduced arm swing and higher asymmetry than healthy controls, although the formula for calculating ASA was slightly different. Furthermore, the results on amplitude and asymmetry are comparable with those reported in [17], although the study’s objectives were different. Regarding spatiotemporal parameters, the measures for the PD group are in line with those of [10]. Unfortunately, this study does not report measures on arm swing, only the effects on spatiotemporal parameters of increasing the amplitude and frequency of arm swing movements. Comparison between the PD and HC groups in [24] shows a statistically significant difference in arm swing parameters, although the different parameters were estimated using an inertial sensor. No study has reported analysis of body sway during walking, although it seems to be an essential gait characteristic to distinguish between post-stroke and PD subjects, as shown in [46].

Referring to the second objective of the study, and specifically the definition of the synchrony indices, it appeared that SI_ARM-LEG_ and SI_ARMS_ were statistically significant in distinguishing the two groups. However, more significant seemed to be the synchrony of arm movements (SI_ARMS_). The alteration of opposite arm movements (i.e., when one moves forward, the other moves backward), quantified by the synchrony index, could indicate the transition from a healthy to a pathological gait. Therefore, monitoring could be helpful for the early identification of a later and more visible disorder manifesting in other aspects of walking.

Regarding the third objective of the study, namely, the correlation between arm swing and spatiotemporal parameters, we found only one study that performed this analysis using the Pearson’s correlation coefficient instead of the nonparametric Spearman’s correlation coefficient, as in our study. Specifically, [11] reported a relationship between arm swing amplitude (as an angular measure) and gait speed and stride length. We confirmed the significant correlation of the angular measures with the speed parameters defined in our study (i.e., STEP_VEL_ and GAIT_VEL_), but we did not obtain a significant correlation with stride length. In contrast, our results suggest that other arm swing parameters strongly correlated with additional spatiotemporal parameters, as shown in Figure 4. This analysis could support the identification of subcategories of gait abnormalities affecting one or more aspects of gait, thus defining different levels of impairment during walking. This evidence is also suggested by the differences in correlation with healthy controls, as shown in Figure 4. Moreover, based on this analysis, it appears that sways of center of mass refer to a specific gait abnormality, uncorrelated with both spatiotemporal and arm swing features. In addition, this analysis could provide new insights into more comprehensive gait assessment, as well as play a preventive role in the risks of “unhealthy” gait, and facilitate the design of specific rehabilitation protocols and remote follow-up of patients.

Overall, preliminary results suggest that the system enables comprehensive and quantitative assessment of walking strategies and characteristics. In addition, some technical features of the system, including its portability, small size, and ease of use, make it suitable for more frequent gait assessments even in unsupervised or poorly supervised settings (such as the home or outpatient clinics). This solution could also facilitate the follow-up on many patients and limit the use of gold-standard systems to only high-risk cases requiring traditional, in-depth gait analysis. Moreover, this solution could support clinicians in monitoring the progression of gait disorders in neurodegenerative diseases (such as PD) and promptly intervene if abnormalities are detected, triggering appropriate treatments, including arm swing enhancement in gait rehabilitation programs, which promote the recovery of safe gait [10,11].

In conclusion, the preliminary results of this study encourage us to continue the analysis of arm swing and correlation with other gait characteristics, but further work remains to overcome some limitations of this study. Indeed, the first step will be to increase the number of subjects involved and analyzed by increasing the reference sample size. This improvement will allow to confirm and consolidate the results obtained in a larger cohort. It will also be interesting to deepen the analysis on groups of PD subjects with different levels of severity, to investigate how the data change in the different classes, whether some of the parameters and indices may already be significant in the early stages of the pathology, and how they vary as a function of pharmacological and rehabilitation treatments, as shown by several studies [16,59]. Similarly, applying the same methodological approach to other pathologies and conditions characterized by impaired walking and movement (for example, in post-stroke subjects) will also be challenging.

In future developments of the study, it is planned to apply machine learning approaches and supervised classifiers according to the three aspects of gait analyzed so far, either by considering them separately or as a whole. From this improvement, it will probably be possible to define a new gait disorder index that considers all three aspects of walking and allows prompt recognition of altered gait with high risk of falls or specific treatments to be activated to recover a safe and effective gait.

## 5. Conclusions

Based on the results presented in this paper, the proposed motion-capture system based on Azure Kinect is capable of quantifying, through objective parameters, arm swing motion during walking in PD subjects, detecting significant differences from control subjects, and obtaining results comparable with other studies in the literature, some of which use a different methodological and technological approach for a different purpose. This ability, together with the already established ability to measure spatiotemporal and center-of-mass parameters, makes the proposed system capable of providing an overall gait assessment concerning its three aspects. This approach is a novelty since studies in the literature generally focus on a single aspect of walking. In fact, to our knowledge, this study is the first in the literature that jointly analyzes aspects related to arm swing, spatiotemporal measurements, and stability during gait using a vision system implemented with the new Azure Kinect.

Finally, the system’s features, including noninvasiveness, ease of use, and portability, make it a suitable tool for monitoring gait alterations on a broader number of subjects and in contexts where traditional gait-analysis systems are not applicable (e.g., home monitoring or in unsupervised environments) or where the accuracy of a gold reference system is not necessary (e.g., for rehabilitation purposes). For these reasons and from the clinical perspective, this approach is particularly promising, as it may pave the way for new patient and disease follow-up strategies using innovative technologies and solutions to support traditional clinical methods.

## Figures and Tables

**Table 1:** Parameters related to arm swing.

Parameter	Meaning	Unit
SWAY_ANT_AP,UD,ML_	Anterior max arm sway ^1,2^	mm
SWAY_POS_AP,UD,ML_	Posterior max arm sway ^1,2^	mm
SWAY_RANGE_AP,UD,ML_	Range of arm sway ^1,2^	mm
PATH_TOT_	Total distance travelled inside GAP ^2^	mm
SWAY_AREA	Area of arm movement (AP-ML) ^2^	mm^2^
SPEED_AP_	Maximum speed on AP ^2^	mm/s
ANGLE_ANT	Max anterior angle ^2^	deg
ANGLE_POS	Max posterior angle ^2^	deg
ANGLE_RANGE	Range of arm angle ^2^	deg
ASA_ANGLE_	Asymmetry of ANGLE_ANT	%
ASA_PATH_	Asymmetry of PATH_TOT_	%
ASA_AP-RANGE_	Asymmetry of SWAY_RANGE_AP_	%
SI_ARM-LEG_	Synchrony index of arm and opposite leg ^2^	-
SI_ARMS_	Synchrony index of arms	-

^1^ On anteroposterior (AP), mediolateral (ML), and up–down (UD) directions. ^2^ Computed separately for left and right arm.

**Table 2:** Spatiotemporal and COM_HIP_ parameters related to gait analysis.

Parameter	Meaning	Unit
STEP_LEN_	Step length ^1^	m
STEP_WIDTH_	Step width ^1^	m
STEP_VEL_	Step velocity ^1^	m/s
STEP_TIME_	Duration of step ^1^	s
STRIDE_LEN_	Length of gait cycle ^1^	m
DOUBLE_SUPP_	Duration of double support ^1^	s
STANCE_DUR_	Duration of stance phase ^1^	% of gait cycle
GAIT_VEL_	Gait velocity	m/s
CADENCE	Gait cadence	steps/min
STEP_NUM_	Number of steps	#
STRIDE_NUM_	Number of strides	#
ML_SWAY_	Mediolateral sway of COM_HIP_	mm
V_SWAY_	Vertical sway of COM_HIP_	mm

^1^ Computed separately for left and right legs.

**Table 3:** Median and quartiles (first and third) relative to arm swing parameters for PD and HC groups, with test statistic, *p*-value, and significance level.

	Median (1st and 3rd Quartiles)		Mann–Whitney		*t*-Test	
**Parameter (unit)**	**PD Group**	**HC Group**	**Statistic**	***p*-Value**	**Statistic**	***p*-Value**
SWAY_ANT_AP_ (mm)	135.22 (75.88, 187.41)	199.49 (160.25, 223.13)	599	<0.001 ***	5.25	<0.001 ***
SWAY_ANT_UD_ (mm)	30.70 (13.85, 43.26)	50.26 (41.40, 57.81)	515	<0.001 ***	5.74	<0.001 ***
SWAY_ANT_ML_ (mm)	19.35 (11.39, 32.45)	33.37 (7.81, 48.16)	1130	0.212	1.39	0.170
SWAY_POS_AP_ (mm)	−41.98 (−110.36, −5.15)	−78.16 (−102.70, −32.25)	1119	0.187	−1.04	0.300
SWAY_POS_UD_ (mm)	−19.82 (−40.20, −5.24)	−27.08 (−40.60, −8.83)	1246	0.629	0.88	0.380
SWAY_POS_ML_ (mm)	−25.53 (−52.57, −12.93)	−29.41 (−56.03, −15.56)	1261	0.700	−0.29	0.770
SWAY_RANGE_AP_ (mm)	162.79 (101.58, 247.12)	278.65 (211.52, 317.21)	714	<0.001 ***	4.14	<0.001 ***
SWAY_RANGE_UD_ (mm)	50.25 (36.07, 64.77)	73.41 (59.63, 89.13)	736	<0.001 ***	2.07	0.004 **
SWAY_RANGE_ML_ (mm)	54.77 (41.02, 73.05)	63.57 (44.87, 80.51)	1077	0.111	1.77	0.080
PATH_TOT_ (mm)	930.32 (602.01, 1404.38)	1237.39 (912.23, 1428.66)	1001	0.036 *	1.41	0.160
SWAY_AREA (mm^2^)	5735.16 (2454.36, 8464.25)	8791.01 (6812.10, 10,292.27)	840	0.002 **	230	0.020 *
SPEED_AP_ (mm/s)	365.01 (231.26, 646.22)	850.81 (533.84, 952.50)	623	<0.001 ***	2.98	<0.001 ***
ANGLE_ANT (deg)	19.29 (8.92, 24.09)	26.39 (21.89, 29.13)	651	<0.001 ***	4.93	<0.001 ***
ANGLE_POS (deg)	−10.33 (−15.40, −6.93)	−10.91 (−13.66, −8.17)	1303	0.911	0.13	0.900
ANGLE_RANGE (deg)	27.33 (15.09, 38.34)	38.24 (31.03, 42.22)	787	<0.001 ***	3.68	<0.001 ***

***: *p*-value < 0.001; **: *p*-value < 0.01; *: *p*-value < 0.05.

**Table 4:** Median and quartiles (first and third) relative to spatiotemporal and COM parameters for PD and HC groups, with test statistic, *p*-value, and significance level.

	Median (1st and 3rd Quartiles)		Mann–Whitney		*t*-Test	
**Parameter (unit)**	**PD Group**	**HC Group**	**Statistic**	***p*-Value**	**Statistic**	***p*-Value**
STEP_LEN_ (m)	0.57 (0.53, 0.63)	0.64 (0.60, 0.70)	622	<0.001 ***	4.35	<0.001 ***
STEP_WIDTH_ (m)	0.14 (0.12, 0.18)	0.12 (0.08, 0.18)	995	0.032 **	−0.333	0.074
STEP_VEL_ (m/s)	0.97 (0.85, 1.11)	1.18 (1.07, 1.27)	609	<0.001 ***	4.27	<0.001 ***
STEP_TIME_ (s)	0.58 (0.53, 0.62)	0.55 (0.53, 0.57)	946	0.014 **	−2.63	0.010 **
STRIDE_LEN_ (m)	1.10 (1.03, 1.25)	1.24 (1.19, 1.37)	644	<0.001 ***	4.36	<0.001 ***
DOUBLE_SUPP_ (s)	0.27 (0.17, 0.37)	0.20 (0.13, 0.24)	840	0.002 **	−3.46	<0.001 ***
STANCE_DUR_ (%)	62.59 (59.67, 65.57)	59.34 (56.48, 61.42)	787	<0.001 ***	−3.83	<0.001 ***
GAIT_VEL_ (m/s)	0.90 (0.81, 1.02)	1.07 (0.99, 1.17)	620	<0.001 ***	4.60	<0.001 ***
CADENCE (steps/min)	109.29 (99.17, 119.00)	111.63 (108.11, 115.38)	1110	0.168	2.49	0.014 *
STEP_NUM_ (#)	2.00 (2.00, 3.00)	2.00 (1.00, 2.00)	870	<0.001 ***	−3.69	<0.001 ***
STRIDE_NUM_ (#)	2.00 (1.00, 2.00)	1.00 (1.00, 1.25)	825	<0.001 ***	−3.96	<0.001 ***
ML_SWAY_ (mm)	57.84 (47.73, 73.16)	54.27 (47.19, 60.86)	286	0.424	−1.06	0.292
V_SWAY_ (mm)	40.62 (29.82, 45.95)	36.33 (32.66, 44.46)	305	0.653	−0.461	0.647

***: *p*-value < 0.001; **: *p*-value < 0.01; *: *p*-value < 0.05.

**Table 5:** Median and quartiles (first and third) relative to asymmetry and synchrony indices for PD and HC groups, with test statistic, *p*-value, and significance level.

	Median (1st and 3rd Quartiles)		Mann–Whitney		*t*-Test	
**Index (unit)**	**PD Group**	**HC Group**	**Statistic**	***p*-Value**	**Statistic**	***p*-Value**
ASA_ANGLE_ (%)	10.48 (3.94, 21.24)	4.47 (2.93, 6.88)	806	<0.001 ***	4.11	<0.001 ***
ASA_PATH_ (%)	11.17 (3.88, 16.79)	5.95 (4.21, 11.10)	984	0.027 *	2.47	0.015 *
ASA_AP-RANGE_ (%)	14.31 (5.91, 18.65)	5.93 (3.93, 8.78)	796	<0.001 ***	4.04	<0.001 ***
SI_ARM-LEG_ (-)	0.79 (0.60, 0.90)	0.89 (0.80, 0.93)	972	0.022 *	2.86	0.005 **
SI_ARMS_ (-)	−0.77 (−0.87, −0.40)	−0.93 (−0.94, −0.82)	574	<0.001 ***	−5.07	<0.001 ***

***: *p*-value < 0.001; **: *p*-value < 0.01; *: *p*-value < 0.05.

**Table 6:** A subset of the most representative parameters for the left and right sides related to the performance shown in Figure 2 (PD group).

	Subject #6 (PD Group)		Subject #10 (PD Group)	
**Parameter (unit)**	**Left**	**Right**	**Left**	**Right**
SWAY_ANT_AP_ (mm)	41.47	61.63	267.42	249.94
SWAY_ANT_UD_ (mm)	−40.16	46.85	27.00	22.70
SWAY_RANGE_AP_ (mm)	295.89	123.95	411.13	395.67
SWAY_RANGE_UD_ (mm)	60.69	84.90	168.41	199.74
PATH_TOT_ (mm)	2522.04	1408.56	1551.77	1575.54
SWAY_AREA (mm^2^)	14,778.15	7287.46	14,328.10	9061.74
SPEED_AP_ (mm/s)	780.29	309.44	1086.99	900.00
ANGLE_ANT (deg)	5.51	9.12	38.13	34.77
ANGLE_RANGE (deg)	40.34	18.06	55.21	52.68
ASA_ANGLE_ (%)	23.24		1.52	
ASA_PATH_ (%)	17.61		0.51	
ASA_AP-RANGE_ (%)	24.78		1.25	
SI_ARM-LEG_ (-)	0.90	0.86	0.90	0.87
SI_ARMS_ (-)	−0.77		−0.91	
**Parameter (unit)**	**Left**	**Right**	**Left**	**Right**
STEP_LEN_ (m)	0.28	0.25	0.82	0.83
STEP_WIDTH_ (m)	0.31	0.28	0.16	0.08
STEP_VEL_ (m/s)	0.45	0.41	1.54	1.59
STEP_TIME_ (s)	0.62	0.62	0.53	0.52
STRIDE_LEN_ (m)	0.49	0.51	1.67	1.57
DOUBLE_SUPP_ (s)	0.86	0.86	0.13	0.13
STANCE_DUR_ (%)	80.34	75.34	59.11	57.89
GAIT_VEL_ (m/s)	0.39		1.47	
CADENCE (steps/min)	99.17		126.32	
STEP_NUM_ (#)	4	4	1	2
STRIDE_NUM_ (#)	4	3	1	1
ML_SWAY_ (mm)	115.24		55.44	
V_SWAY_ (mm)	28.41		66.55	

**Table 7:** A subset of the most representative parameters for the left and right sides related to the performance shown in Figure 3 (HC group).

	Subject #3 (HC Group)		Subject #5 (HC Group)	
**Parameter (unit)**	**Left**	**Right**	**Left**	**Right**
SWAY_ANT_AP_ (mm)	150.43	137.53	195.53	225.63
SWAY_ANT_UD_ (mm)	37.39	37.40	53.14	63.74
SWAY_RANGE_AP_ (mm)	279.31	136.28	333.67	327.56
SWAY_RANGE_UD_ (mm)	61.11	45.45	62.55	59.31
PATH_TOT_ (mm)	1330.83	618.68	1455.22	1465.84
SWAY_AREA (mm^2^)	8264.99	3076.61	7416.28	13,192.44
SPEED_AP_ (mm/s)	829.42	466.92	927.41	951.26
ANGLE_ANT (deg)	23.15	17.38	27.09	26.80
ANGLE_RANGE (deg)	39.48	20.47	43.50	46.18
ASA_ANGLE_ (%)	19.59		1.92	
ASA_PATH_ (%)	22.33		0.26	
ASA_AP-RANGE_ (%)	21.14		0.61	
SI_ARM-LEG_ (-)	0.89	0.92	0.94	0.86
SI_ARMS_ (-)	−0.94		−0.91	
**Parameter (unit)**	**Left**	**Right**	**Left**	**Right**
STEP_LEN_ (m)	0.59	0.64	0.73	0.75
STEP_WIDTH_ (m)	0.20	0.07	0.24	0.10
STEP_VEL_ (m/s)	1.04	1.16	1.28	1.27
STEP_TIME_ (s)	0.57	0.55	0.57	0.59
STRIDE_LEN_ (m)	1.19	1.21	1.42	1.48
DOUBLE_SUPP_ (s)	0.27	0.25	0.17	0.21
STANCE_DUR_ (%)	61.46	63.21	56.03	58.04
GAIT_VEL_ (m/s)	0.93		1.15	
CADENCE (steps/min)	110.09		107.14	
STEP_NUM_ (#)	2	2	2	1
STRIDE_NUM_ (#)	2	1	1	1
ML_SWAY_ (mm)	75.58		54.48	
V_SWAY_ (mm)	32.49		73.66	

## Data Availability

Data are available on request.
